# Severe Hyponatremia-Induced Stress Cardiomyopathy: A Case Report and Review of Literature

**DOI:** 10.1155/2020/2961856

**Published:** 2020-03-31

**Authors:** Irushna Perera, Sanjeewa Rajapakse, Shamila T. De Silva

**Affiliations:** ^1^Colombo North Teaching Hospital, Ragama, Sri Lanka; ^2^Faculty of Medicine, University of Kelaniya, Sri Lanka

## Abstract

Takotsubo or stress cardiomyopathy is a non ischemic disease affecting the myocardium, which presents with typical features of myocardial ischemia. Although the presentation with acute central chest pain and shortness of breath mimics acute myocardial ischemia, there is an absence of actual disruption of cardiac blood supply via the coronaries due to acute plaque rupture or vascular spasm. The underlying pathophysiology of this clinical entity remains largely unclear, but a definite association with physical or emotional stress has been well established, hence the term “stress cardiomyopathy.” The list of potential triggers continues to grow as the disorder is increasingly detected by clinicians and cardiologists, with better clinical insight and improved availability of cardiac investigations. We report a patient with Takotsubo cardiomyopathy associated with severe hyponatremia.

## 1. Introduction

Takotsubo cardiomyopathy is a potentially reversible disorder affecting the myocardium. It is characterized by transient apical ballooning of the left ventricle, evident on transthoracic echocardiography. Typically, the disease is seen in postmenopausal women, who present with central tightening chest pain and shortness of breath, with electrocardiographic changes which mimic coronary artery disease and a mild elevation of cardiac enzymes [1]. The condition is usually benign and has a self-limiting course, with recovery within days to weeks following removal of the emotional or physical trigger. Rarely, the syndrome may be complicated by lethal ventricular arrhythmias or ventricular rupture [2].

## 2. Case Report

A 73-year-old woman was admitted to the Emergency Department with vomiting, diarrhea, and confusion for two days. She had been generally unwell during the preceding two weeks, with poor oral intake. She had also developed a few presyncopal episodes during this time, but she had not lost consciousness. She denied chest pain or fever. She was on treatment for hypertension with verapamil, losartan, and spironolactone, along with atorvastatin for elevated lipids.

**Figure 1:**
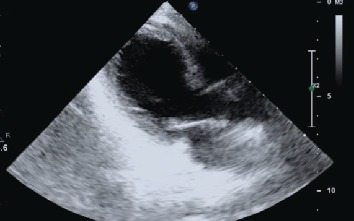
Echocardiogram long axis view revealed apical ballooning of the LV.

On admission, she was unwell, with a heart rate of 100 beats/minute, blood pressure of 140/90 mmHg, peripheral oxygen saturation of 97% breathing ambient air, and respiratory rate of 25 breaths/minute. She did not appear dehydrated. Electrocardiogram revealed ST segment elevations in leads I, II, aVF, and V3 to V6. A cardiac biomarker assay revealed a Troponin I of 0.343 ng/ml (upper limit of reference 0.04 ng/ml). She was initiated on treatment for an acute coronary event, with loading doses of oral aspirin, clopidogrel, atorvastatin, and subcutaneous low molecular weight heparin. Echocardiography revealed apical ballooning of the left ventricle (Figure 1) with mild left ventricular systolic dysfunction, suggestive of stress cardiomyopathy. However, treatment of a presumed acute coronary event was continued.

She was severely hyponatremic, with serum sodium of 104 mmol/L. Serum potassium was 3.7 mmol/L, and serum chloride was 72 mmol/L. As the patient was symptomatic, serum sodium was corrected slowly, with 3% saline infusions over a few days. Concurrently, all medications liable to cause sodium depletion were withheld.

The day after admission, the patient developed a transient drop in blood pressure, which was managed with inotrope support for 18 hours, following which her hemodynamic status remained stable.

With the slow correction of serum sodium and treatment of a presumed acute coronary event, the patient's general condition improved, but she continued to have a residual amount of confusion. A psychiatry consultation was requested, and a diagnosis of a mixed anxiety, and depressive disorder was made. However, no drug treatment was given for her psychiatric illness, since the consulting psychiatrist felt counseling alone would be sufficient treatment.

**Figure 2:**
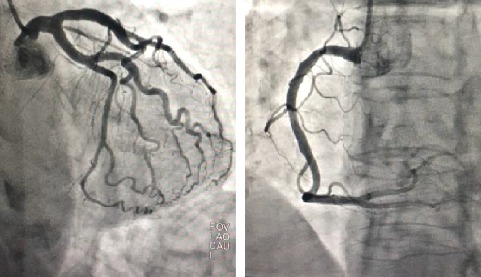
Coronary angiogram revealed normal coronary arteries.

**Figure 3:**
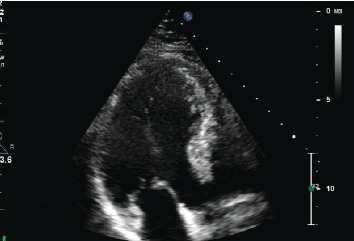
Subsequent echocardiogram revealing a completely recovered myocardium.

A coronary angiogram was performed which failed to reveal any obstruction to the coronary blood flow (Figure 2), and a subsequent echocardiogram revealed a completely recovered myocardium with a left ventricular ejection fraction of 60% (Figure 3). These finding make stress cardiomyopathy the most likely diagnosis, possibly triggered by severe hyponatremia.

Investigations to identify the cause of hyponatremia revealed a serum osmolality of 234 mOsm/L, a urinary osmolality of 597 mOsm/L, and a urinary sodium excretion of 145 mmol/L. There was no evidence of renal or hepatic derangement, and thyroid functions were normal, making the syndrome of inappropriate ADH secretion (SIADH) a possible cause for hyponatremia. However, as the patient was on long-term angiotensin converting enzyme inhibitors and spironolactone for hypertension, a complex interplay of mechanisms could possibly have resulted in severe hyponatremia. Over the next six months at follow-up visits, the patient remained well, with normal serum sodium levels.

## 3. Discussion

Takotsubo cardiomyopathy is a syndrome characterized by transient, regional, systolic dysfunction of the left ventricle, mimicking regional wall motion abnormalities seen in coronary artery disease, but with absence of angiographic evidence of coronary artery obstruction [3]. The entity is also known as the apical ballooning syndrome due to its characteristic appearance on echocardiography and left ventriculography. Takotsubo in Japanese means “a fishing pot for trapping octopus,” similar in shape to the left ventricle in patients with this condition [4]. Usually, the regional wall motion abnormality extends beyond the territory perfused by a single coronary artery [5].

The pathophysiology of Takotsubo cardiomyopathy remains poorly understood, even though numerous theories on its pathogenesis have been proposed. One of the postulated mechanisms is a surge of catecholamines in the circulation resulting in a stunned myocardium, microvascular dysfunction, and spasm of coronary arteries [1]. The proposed role of a catecholamine surge in the development of Takotsubo cardiomyopathy has been observed where cardiomyopathy has developed with infusions of noradrenaline and dopamine, initiated for causes other than cardiogenic shock [6, 7]. Limited data is available on possible triggers, even though the list keeps growing with increasing recognition of the disorder. Commonly, the onset follows intense emotional or physical stress. However, a few cases of Takotsubo cardiomyopathy associated with endocrine abnormalities and electrolyte disturbances have been described, where hyponatremia-associated Takotsubo cardiomyopathy has been reported [8–10].

Hyponatremia is a common electrolyte disorder, encountered especially among elderly patients, in whom finding the etiology of hyponatremia remains challenging as most often it tends to be multifactorial [7]. However, prescription drugs for various comorbidities, especially in the elderly, is the usual culprit, as was observed in our patient as well. The drugs with established potential to cause hyponatremia are diuretics, antidepressants, antipsychotics, and anticonvulsants, resulting in a significant number of Takotsubo cardiomyopathy cases being detected in patients on treatment for hypertension or heart failure and among those in psychiatry wards [8]. Development of Takotsubo cardiomyopathy, secondary to hyponatremia resulting from prescribed drugs, is well recognized. As with the well-known sequelae of hyponatremia, the rapidity with which the electrolyte disturbance develops is likely to be a deciding factor in the occurrence of Takotsubo cardiomyopathy, as chronic, persistent hyponatremia is a less likely trigger [9]. The mechanism of transient cardiac dysfunction in the setting of hyponatremia, whatever the etiology may be, remains unclear, but this association has been previously observed [10–13]. The possible mechanisms proposed are central nervous system dysfunction secondary to hyponatremia, which results in myocardial injury due to raised catecholamine levels, interference of myocardial contractility by low sodium concentration due to disruption of myocyte sodium and calcium exchange, and swelling of cardiac myocytes due to the resultant hypotonicity [9].

The case presented demonstrates the possible association of hyponatremia with stress cardiomyopathy, where the patient made a complete recovery from the cardiac disorder with correction of the electrolyte disturbance. As the detection rate of Takotsubo cardiomyopathy increases with increasing availability of sophisticated cardiac investigations, its possible triggers need to be kept in mind to guide clinicians towards an early and accurate diagnosis.
